# Self-cleaning of a hydrophobic surface by a rolling water droplet

**DOI:** 10.1038/s41598-019-42318-3

**Published:** 2019-04-05

**Authors:** Ghassan Hassan, Bekir Sami Yilbas, Abdullah Al-Sharafi, Hussain Al-Qahtani

**Affiliations:** 1 Department of Mechanical Engineering, King Fahd University of Petroleum and Minerals (KFUPM), Dhahran, 31261 Saudi Arabia; 2 Center of Research Excellence in Renewable Energy (CoRE-RE), King Fahd University of Petroleum and Minerals (KFUPM), Dhahran, 31261 Saudi Arabia; 3 Researcher at K.A.CARE Energy Research & Innovation Center at Dhahran, Dhahran, Saudi Arabia; 4 Senior Researcher at K.A.CARE Energy Research & Innovation Center at Dhahran, Dhahran, Saudi Arabia

## Abstract

A water droplet behavior on a hydrophobic surface is examined relevant to the dust particles removal from the surface. Surface crystallization of polycarbonate is realized in acetone bath and the resulting surface is coated by the functionalized nano-size silica particles towards reducing contact angle hysteresis. This arrangement provides droplet rolling/sliding on the hydrophobic surface. Droplet translational velocity is formulated and predictions are compared with those resulted from the high speed recorded data. Influence of surface inclination angle on droplet dynamics is investigated and the dust removal mechanism on the inclined surface is analyzed. It is found that predictions of droplet translational velocity agree well with those obtained from the experiment. Droplet rolling dominates over sliding on the inclined surface and droplet sliding velocity remains almost 10% of the droplet translational velocity. The main mechanism for the dust particles removal is associated with the droplet fluid cloaking of the dust particles during its transition on the hydrophobic surface. Droplet acceleration, due to increased surface inclination angle, has effect on the rate of dust particles removal from the surface, which is more apparent for large droplet volumes. Increasing droplet acceleration improves the coverage area of the clean surface.

## Introduction

Dust storms have significant impact on environment while causing environmental changes around the Globe^1^. The activity of the dust particles around the Globe is mainly governed by modification of desert surfaces by vehicular traffic, distortion of vegetation area by natural disasters, and climate change by global warming under human influence^2^. Minimization of the influence of the dust particles and measures for dust mitigation become one of the the recent challenges in scientific research. Dust removal from surface of energy harvesting devices, such as photovoltaics and solar concentrated troughs, remains vital for sustainable operation, particularly in close regions of the desert environment. Several methods have been proposed for dust removal from surfaces^3–12^ and some of these include mechanical brushing^3–5^, air jet blowing^6^, electrostatic repelling^7,8^, water film washing and splashing^9,10^, water droplet cleaning^11,12^. Some of these surface cleaning methods are involved with energy intensive processes and required external cleaning resources such as compressed air and clean water. Self-cleaning of surfaces provides several advantages over the conventional water film/jet or compressed air jet cleaning methods such as low energy and minimum resource requirements. However, in self-cleaning applications, a prior treatment of surface is required towards achieving the texture characteristics with a hydrophobic wetting state. The topology of texture with hierarchical distributed micro/nano pillars are favorable for the hydrophobic wetting state; in which case, air gaps captured in between the texture pitches limits the interfacial contact area between the solid and the liquid surfaces while enhancing liquid contact angle on the surface. Although contact angle plays a major role for spherical droplet forming on the hydrophobic surfaces, contact angle hysteresis becomes vital for the droplet mobility on the hydrophobic surfaces. In general, droplet in motion experiences rolling/sliding on the hydrophobic surfaces. The droplet size, acceleration, and wetting state of the surface play a major role on the droplet dynamics. The hydrophobic surface with low contact angle hysteresis droplet suffers from rolling rather than sliding^13^. In addition, small size droplets roll like marbles and they do not suffer from wobbling on the surface unlike large size droplets; in which case, gravitational influence causes droplet bulging and wobbling during rolling^11^. Depending on the contact angle hysteresis, retaining force effects the droplet rolling and droplet undergoes mixed motion consisting of sliding and rolling on the surface for large values of contact angle hysteresis. With the dust particles presence on the hydrophobic surface, droplet retaining force increases and droplet motion is governed by both mixture of sliding and rolling. Water droplet picks up the dust particles from the surface during rolling/sliding on the hydrophobic surface; however, the rate of the dust particles picking depends on the droplet size and the dust particles cloaking by the droplet liquid. However, the dust particles cloaking involves with the residence time of droplet on the surface during sweeping^14^. Hence, the droplet acceleration influences the rate of picking the dust particles by the droplet. Consequently, investigation of the mechanisms for the droplet-dust particles interactions on the hydrophobic surfaces remains essential for self-cleaning of surfaces and exploring the effect of droplet acceleration on the dust particles removal rate becomes critical.

Self-cleaning applications of surfaces have been investigated earlier while mimicking the nature^15–20^. In general, the surfaces of lotus leaves, rice rusks, and rose petals have self-cleaning characteristics, which also have the hydrophobic wetting state on the surface. Hence, the hydrophobic state remains critical for self-cleaning applications. Several methods have been introduced to create hydrophobic and superhydrophobic surfaces for self-cleaning abilities. Microstructure casting of chemically textured surfaces towards achieving hydrophobic surfaces becomes interesting for various self-cleaning applications, such as repelling of Portland cement from surfaces^15^. Inspiring the nepenthes pitcher towards fabricating anti‐wetting coatings became successful via using the combination of fluoro‐silicone nanofilaments (fluoro‐SNs) and Krytox liquids, perfluoropolyethers^16^. Ones-step texturing of surfaces by a laser treatment could also create hydrophobic wetting state on the surface, which could be used for self-cleaning purposes^17,18^. One of the challenges of the hydrophobic surfaces is the durability of texture topology over the long period of usage in self-cleaning applications; however, recent developments in surface texturing processes enable to produce such surfaces. The method of electrodeposition of Zn/ZnO layer could possibly result in durable hydrophobic surface with self-cleaning ability^19^. Chemical etching of galvanic alloy, such as Ag-Fe_3_O_4_/Fe, could result in durable hydrophobic surfaces. The processes followed by oxidation, lauryl mercaptan modification, and compression molding provide hydrophobic wetting state on copper alloys^20^. The attainment of the hydrophobic polycarbonate surface via nitric acid treatment and followed by subsequent surface silylation through methyltrichlorosilane (MTCS) remained interesting for self-cleaning of decorative surfaces^21^. The electroless copper plated processing of wood surfaces provides self-cleaning characteristics together with electromagnetic shielding, which could be used in some applications of electrical and electronic circuits where the cleaning of surfaces are required. However, research into the cost effective and environmentally friendly processes for surface texturing towards achieving hydrophobic wetting state in use of self-cleaning applications is still progressing. Moreover, the droplet dynamics on hydrophobic surfaces remains critical for self-cleaning of particles on the surfaces. Rolling and sliding are the basic mechanisms governing the droplet dynamics on the hydrophobic surfaces^22^. Inclination of the surface increases the gravitational influence on the droplet mass while causing droplet acceleration along the inclination direction. The mode of occurrence of rolling and sliding depends on the force balance between pinning and gravitation^22^. However, the dynamics of droplet adhesion change on the surface and droplet acceleration can vary along the surface^23^. The texture morphology of the surface plays important role on the droplet dynamics; hence the micro-pillar type texture remains critical for the mode of droplet dynamic behavior^24^ and ordered nanoball matrix fluorocarbon structures can result in different droplet behavior in terms of rolling/sliding on the surface^25^. The direction of rolling droplet can be controlled by creating the well-designed asymmetric surface texture morphology^26^. Alternatively, exchanging of the wetting condition on the surfaces significantly influences the mode of droplet motion. This remains particularly interesting when the surface is coated by the phase change material^27^. In this case, droplet pinning on hydrophobic surface replaces with sliding motion due to the liquid layer formed on the surface. However, upon solidification of the phase change material, droplet rolling takes over on the surface^27^. Thermocapillary effect contributes to the droplet mobility because of flow forces generated in the fluid^28^. One of the ways generating thermocapillary forces in droplet fluid is local radiative heating of the droplet surface; in which case, it is possible to generate fluid forces overcoming the adhesion force on the surface^28^. The fundamental understanding the droplet dynamics on the hydrophobic surfaces significantly contributes to enhancement of the surface texture design towards improving self-cleaning ability of the hydrophobic surfaces.

Dust deposition on surfaces alters the physical properties of surfaces, such optical transmittance and absorption, and modifies the texture morphology, which becomes significantly important in humid air ambient^29^. Water condensation on the dust particles can cause dissolution of some dust compounds in water and forms a chemically active liquid on the surface^30^. In some cases, this gives rise permanent damage on the texture and alters the surface properties^31^. Moreover, investigation of the mechanism of the dust particles deposition on surfaces becomes important towards minimization of particle accumulation, which becomes critical for sustaining the high performance of solar energy harvesting devices^32,33^. The right estimation of the cleaning frequency of the surfaces of the solar energy harvesting devices, such as photovoltaics and solar concentrated receivers, is essential to reduce the cleaning costs^33,34^. The best cleaning frequency could be set when the device output power is reduced by 5%^33^. The wind velocity also influences the dust accumulation; hence, it alters the cleaning frequency of the surfaces, which depend on the wind direction and intensity^35^. The dust particles with sizes greater than 1 µm can be removed from the surface by the wind power; however, small size particles require effective cleaning procedure to remove them from the surfaces^35^. The dust particles contribute absorption and reflection of the incident solar irradiation while causing a local temperature increase on the device surface^36^. This alters the device operational efficiency and lowers the device output power^37^.

Although droplet dynamics on soiled hydrophobic surfaces was studied earlier^11,12^, the influence of droplet acceleration on the dust particles removal rate from the surface is left for future study. The droplet acceleration on the inclined hydrophobic surface is important in terms rolling, sliding, and puddling of the droplet. The droplet mobility on the hydrophobic surfaces affects the amount of dust particles picked up. This is because of the cloaking of the dust particles by the droplet fluid. The transition time, at which the duration of the droplet wetting length sweeps along the soiled surface, must be comparable to the cloaking time of the dust particles by the droplet fluid on the surface^12^. Hence, the transition time of the droplet sweeping over the soiled surface becomes short as the droplet accelerates. The dust particles, which are cloaked by the droplet fluid, can be removed. Consequently, the droplet acceleration can influence the dust particles removal rate from the hydrophobic surfaces. In this study, the influence of droplet acceleration on the removal of the dust particles is considered and droplet dynamics in terms of rolling and sliding are analyzed. The rate of dust particles removal is estimated and dust particles residues on the droplet path are analyzed using energy dispersive spectroscopy and scanning electron microscopy. The droplet acceleration is introduced via varying the inclination of the hydrophobic surface and particle cloaking time is compared with the droplet transition time.

## Experiment

### Texturing and hydrophobization of polycarbonate surface

A polycarbonate wafer was prepared in 25 mm × 250 mm × 2.5 mm (width × length × thickness) dimension. The wafers were crystallized incorporating acetone immersion for 4 minutes. Acetone concentration was kept at 60% (by volume in water) in line with the early study^38^. The selection of immersion time and acetone concentration relied on several tests such that crystallization parameters resulting in hierarchical textures without defect sites, such as voids and cracks on the surface, were selected. The contact angle measurements were carried out on the crystallized surfaces. Goniometer measurements demonstrated that crystallized surface was hydrophobic with the contact angle of about 136° ± 4° and the contact angle hysteresis was about 38°. Hence, resulting surface did not create the lotus effect for the droplet mobility rather the droplet adhered onto the surface. In order to create a lotus effect on the crystallized surface, the surface was coated by functionalized nano-size silica units via adopting the deep coating technique. Hence, the silica nanoparticles were synthesized adopting the technique proposed in the early study^39^. Tetraethyl orthosilicate (TEOS), isobutyltrimethoxysilane (OTES), ethanol, and ammonium hydroxide were incorporated in the synthesizing cycle. In this cycle, tetraethyl orthosilicate (TEOS), isobutyltrimethoxysilane (OTES), ethanol, and ammonium hydroxide were utilized. Hence, 14.4 mL of ethanol, 1 mL of ultrapure water, and 25 mL of ammonium hydroxide were stirred for 15 minutes obtaining the mixture. TEOS (1 mL) was diluted with ethanol (4 ml). The diluted TEOS was added to the prepared mixture and it was stirred for 25 minutes. The modified silane molecule was added in a molar ratio of 3:4 to the final mixture and it was, then, stirred for 10 hours at room temperature. The solvent casting was applied to deposit the final mixture solution onto the substrate surfaces. Later, substrate surfaces were centrifuged and washed with ethanol for removal of reactants. The contact angle of the coating was measured and the results demonstrated that the contact angle of 158° ± 2° with hysteresis of 2° ± 2° was achieved. This arrangement provided droplet rolling/sliding on the resulting surfaces.

### Tools incorporated for assessment of surface characteristics

A JEOL 6460 SEM (scanning electron microscope) with magnification of 10× to 300 000× was utilized for surface micrography. Atomic force microscopy (AFM/SPM) in contact mode was used to assess the surface topology. X-ray diffraction (D8 Advanced diffractometer, Bruker) with CuKα source was used for the compound examination of the dust particles. The droplet contact angle of surfaces was measured suing goniometer while adopting the method introduced in the early study^40^. A high-speed monitoring system (SpeedSense 9040, Dantec Dynamic) was incorporated to record the droplet dynamics on the surface. A fixture was designed and used to alter the inclination of the hydrophobic surface with in 0° to 10°.

### Collection of dust particles and characterization

The dust particles were collected from the cover glass of photovoltaic panels located in King Fahd University of Petroleum and Minerals in the Kingdom of Saudi Arabia. The dust particles were collected by using the soft brushes and, later, kept in air-tight small containers. The dust particles were initially assessed by size, weight, elemental composition, and shape. The dust particles settled on the surface of the cover glass over one month period was about 20 g/m^2^. However, this amount changed almost 17% (by weight) over six months depending on the wind speed and its direction, which was almost 4 m/s in average.

## Results and Discussion

Dust removal from the inclined hydrophobic surface via droplet rolling/sliding is considered and the effect of droplet acceleration on the dust particles removal rate is investigated. The findings are discussed in accordance with: i) dust particles and characteristics, ii) texture characteristics of hydrophobic surface, and iii) droplet dynamics on inclined hydrophobic surface prior and after dust deposition.

### Dust particles and characteristics

**Figure 1:**
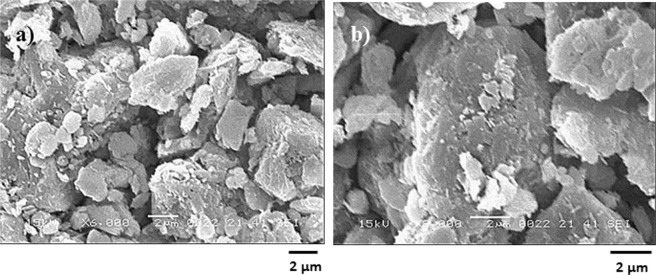
SEM micrographs of dust particles: (**a**) dust particles with various sizes, and (**b**) small dust particles attaches onto large dust particle surface.

**Figure 2:**
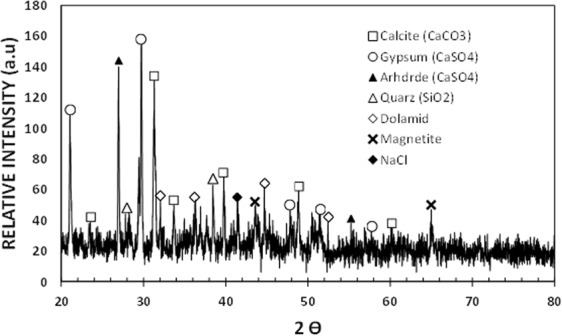
X-ray diffraction of dust particles.

**Figure 3:**
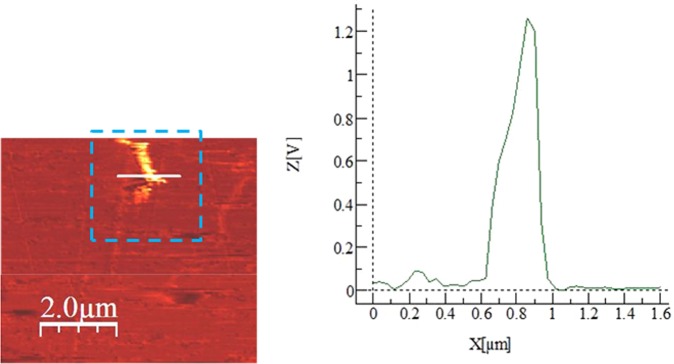
AFM friction data of dust particle. Blue square shows force data due to dust particle on the surface and Z, X graph shows the applied force by AFM prove to move dust particles on the surface.

**Figure 4:**
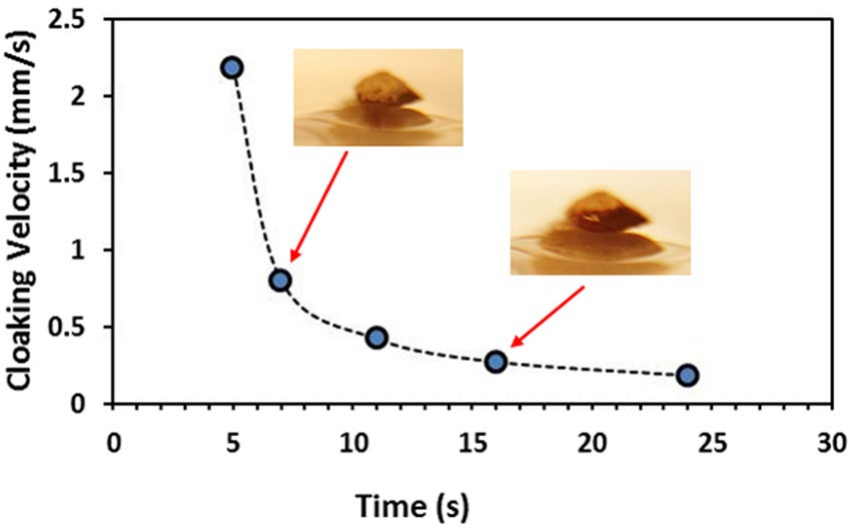
Temporal variation of cloaking velocity of water onto dust particle.

The dust particles are collected form the Dhahran area in the Northern Province of Saudi Arabia. Figure 1 shows SEM micrographs of the dust particles, which consist of various sizes (Fig. 1a) and shapes. The average size of the dust particles is in the order of 1.2 µm, which is consistent with the findings of the early work^38^. The particle size analysis reveals that the dust particles size mainly differs within range of 0.1 µm to 25 µm. However, small size dust particles attach to the large size particles (Fig. 1b). The attachment of the small size dust particles is related to long residence duration of these particles in the near sea atmosphere and solar radiation interaction with the dust particles in the atmosphere can cause ionic charges on the small particles. Upon settlement on the solid surface, these particles attach to the large particles^38^. The dust particles density varies in between 1800 kg/m^3^ to 3600 kg/m^3^ depending on the elemental composition. In general, small size dust particles (≤2 µm) have low density range (1800 kg/m^3^ to 2200 kg/m^3^). The elemental composition of the dust particles is provided in Table 1. The dust particles contain different elements including, Si, Ca, Na, K, Mg, S, O and Cl. The concentration of Cl remains slightly higher for small size dust particles (2 µm ≤). Since the stoichiometric ratio of Cl with Na, K, and Mg does not satisfy the elemental ratio for the salt crystals, Cl appears as dissolved in the particles than forming the crystal structure. The shape of the dust particles is assessed introducing the shape factor. The shape factor is related to the major to minor axis ratio, which is the best fit to the particle, i.e. ${R}_{Shape}=\frac{{P}^{2}}{4\pi A}$, here *P* corresponds to the dust particle perimeter and *A* is the maximum cross-sectional area of the particle. Although optical images of the dust particles are used when obtaining the perimeter and cross-sectional area of the dust particle, the error involved with the estimation is over 30%. Nevertheless, the shape factor gives useful relation about the geometric feature of the dust particles. *R*_*Shape*_ for small size particles (≤2 µm) is almost one and the median of *R*_*Shape*_ is about 2.8 for large particles (≥8 μm). The small particles shape factor (2 μm ≤) becomes almost unity; however, the median of the shape factor for the particles having the size ≥10 μm becomes almost 3. Consequently, depending on the size of the particles, the geometric feature changes considerably. Hence, small size particles appear to be more round than those of large particles, which can be connected to the time evaluation of the particles with small sizes. Figure 2 shows X-ray diffraction pattern of the dust particles. Many crystal peaks appear in the diffraction pattern. The iron peak almost overlaps with the aluminum and silicon peaks. The sulfur peak is due to calcium; hence, anhydrite or gypsum components (CaSO_4_) are present in the particles. Iron is because of clay-aggregated hematite (Fe_2_O_3_). These findings are consistent with the data reported in the early work^38^. The dust particles retention (adhesion) on the surface is obtained from the atomic force microscopy data. Figure 3 shows the adhesion force due to the dust particle on the hydrophobic surface, which is obtained from atomic force microscopy data. Since the retention force is related to the probe deflection of the atomic force microscope in the form of as $F=k\sigma {\rm{\Delta }}V$^41^, where *k* represents the spring constant of the AFM probe tip, *σ* corresponds to *Δz*/*ΔV* (m/V), and *ΔV* is the data (voltage) recorded along the scanned surface when AFM tip is the friction mode. Figure 3 shows the adhesion force data resulted from AFM probe. The data from AFM for the measurement is *k* = 0.12 N/m and Δz/ΔV = 1.481 × 10^−6^ m/V. Hence, the adhesion force for a single dust particle with a size of about 2 μm on the hydrophobic surface is about 35 nN; however, the retention force increases over 60 nN for the particle size of 10 μm. On the other hand, the retention force of a dust particle located on a normal glass surface is well above 80 nN for the dust particle of about 2 μm size while it is about 125 nN for 10 μm size. Hence, joining between the dust particle and the surface remains less than that of the normal glass surface. This behavior is due to the reduced contact area of the particle on the hydrophobic surface because of the airgaps captured in between the texture heights on the surface. Water cloaking of the dust particles is also important for removal of the particles by a rolling/sliding droplets^12^. The liquid cloaking (liquid spreading on surface under interfacial tension) occurs through the stages. Initially, the force equilibrium due to interfacial shear between the liquid and solid and surface tension forms a single-layer of liquid on the solid surface of the particle. Later, spreading of liquid on solid surface takes place while satisfying the Joos’ law^42^. The liquid velocity associated with spreading (*V*_*sp*_) is in the form of ${V}_{sp}\propto {(3{S}_{ow(a)}/4\sqrt{{\mu }_{o}{\rho }_{o}})}^{1/2}{t}^{-1/4}$, here μ_o_ is the liquid viscosity, ρ_o_ is the liquid density, and S_ow(a)_ represents the spreading coefficient of the liquid^43^. The force due to dissipation of liquid energy under the spreading on solid surface is related to the Ohnesorge number $(Oh={\mu }_{o}/\sqrt{{\rho }_{o}a{\gamma }_{oa}})$, here ${\gamma }_{oa}$ is the surface tension of liquid and *a* corresponds to the size of the particle^43^. After adopting the dust particle size ≥0.2 µm, the Ohnesorge number attains values greater than unity (*Oh* > 1). Hence, the large dissipation force is resulted when water cloaks the particle. However, the rate of cloaking of liquid is related to the time for cloaking, which is ∼*k*_*m*_*t*^*1/4*^, here; *k*_*m*_ represents the cloaking factor^43^. Nevertheless, the cloaking takes place for *k*_*m*_*t*^*1/4*^ > 1^44^. Figure 4 shows water cloaking time for the dust particles obtained from the high speed recording data. The water cloaking velocity is about 0.2 mm/s in the early cloaking time while the cloaking time becomes 0.000191083 s for 20 µm dust height in this cycle. However, the cloaking velocity is related to inverse of time of cloaking, which is ∼*Ct*^*−0.5*^, here *C* remains constant, which changes with the particle geometric features, and *t* corresponds to the time for cloaking. Consequently, the dust particles can be cloaked by the rolling/sliding droplet as the transition time of droplet on the hydrophobic surface becomes less than the time for cloaking. Here, the transition time covers the period when the wetted length of the droplet is completely sweep over the hydrophobic surface.

**Table 1:** Elemental composition of dust (wt.%) determined by energy dispersive spectroscopy (EDS).

	Si	Ca	Na	S	Mg	K	Fe	Cl	O
Size ≥ 1.2 μm	12.4	8.4	2.3	1.2	2.5	0.8	1.1	0.6	Balance
Size < 1.2 μm	10.2	7.3	2.8	2.2	1.4	1.5	1.1	1.3	Balance
Dust Residues	12.5	10.1	0.2	2.9	0.4	0.3	1.6	0.1	Balance

### Texture characteristics of hydrophobic surface

**Figure 5:**
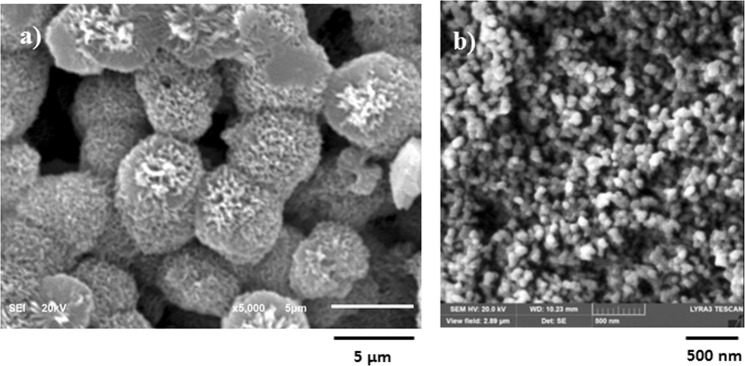
SEM micrographs of crystallized and functionalized silica particles coated surface: (**a**) crystallized polycarbonate surface, and (**b**) functionalized silica particles coated crystallized surface.

**Figure 6:**
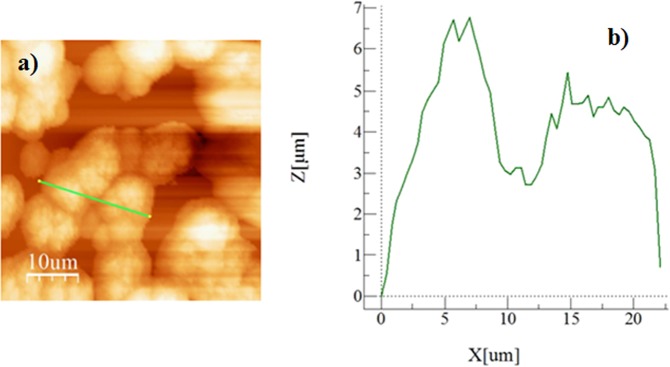
AFM micro-image of silica particles coated crystallized surface and line scan: (**a**) topology of crystallized surface, and (**b**) line scan along the crystallized surface (green line on micro-image shows scanning length).

Figure 5 shows crystallized surface micrographs (SEM images) before and after functionalized nano-sized silica particles (units) coating on the crystallized surface. Crystallized surface demonstrates hierarchical distribution of globules and emanating nano-fibrils like texture from the globules surfaces (Fig. 5a). Similar texture characteristics are reported in the early study^38^. The nano-size units deposited surface demonstrates clusters-like texture characteristics (Fig. 5b) because of agglomeration of silica units, which has about 30 nm diameter. The clustering of these units takes place because of the followings: (i) during synthesizing cycle, the condensing monomer can grew at a higher rate than the rate of nucleation while giving rise to agglomeration of silica units^38^, and (ii) modified silane can results in side-reactions while condensing on the surface of the silica units contributing to clustering of the nano-size units. Small size few voids-like textures are also apparent and these are arbitrarily located on the surface. Figure 6 shows AFM scan along the nano-size units deposited surface. The closely situated globules like texture result in closely spaced peaks in the diagram (Fig. (6)). The roughness of the nano-size silica units coated crystallized surface is about 4.2 µm. Since the diameter of the units is about 30 nm (Fig. 5b), the clustered nano-size units do not significantly contributed to the roughness of the surface. The roughness parameter, which is the area covered by globules over its projected area, is determined from AFM data, and it is about 0.52. Moreover, the contact angle measurements are carried out on the nano-size units coated surface while adopting the technique proposed in the early study^40^. The droplet contact angle is about 158° ± 2° with hysteresis of 2 ± 2° across the entire coating surface. Hence, coating of the surface by nano-size units gives rise to almost uniform wetting state with extremely small hysteresis.

### Dynamics of droplet on inclined hydrophobic surface prior and after dust deposition

Droplet motion on the hydrophobic inclined surface is governed by the force equilibrium along the surface. The retention force due to droplet adhesion, frictional force at the liquid interface, air-drag, and gravitational force are the main driving forces for the droplet motion. The droplet contact angle hysteresis becomes critical for droplet adhesion on the hydrophobic surface while the frictional force is related to the interfacial shear generated across the fluid and hydrophobic surface. The air-drag force is related to the air density, droplet transverse velocity, and droplet diameter. The droplet acceleration on the surface is formulated previously^12^; hence, the governing equation is presented briefly herein. The equation for droplet acceleration is^12^:

1 $$mg\,\sin \,\delta -{F}_{ad}-{F}_{\tau }-{D}_{a}=\frac{2}{5}mR{\omega }^{2}$$

here, *m* corresponds to the droplet mass, *δ* represents the hydrophobic surface inclination angle, *F*_*ad*_, *F*_*τ*_, and *F*_*f*_ are the retention, shear, and frictional force at the surface and the droplet interface, respectively, *D*_*a*_ represents the force due to air-drag, *R* corresponds to the droplet radius, and *ω* is the droplet rotation speed. However, the retention force under the surface tension can be written as^11^:

2 $${F}_{ad}=\frac{24}{{\pi }^{3}}{\gamma }_{LV}fD(cos{\theta }_{R}-cos{\theta }_{A})$$

here, ${\gamma }_{LV}\,\,$represents the surface tension, *f* is the solid fraction (solid-liquid contact fraction because of surface texture), *D* represents the diameter of the droplet (it is almost same area of an ellipse), *θ*_*R*_ is the receding angle, and *θ*_*A*_ corresponding to the advancing angle. The frictional force (shear force at interface) can be written as:

3 $${F}_{\tau }={A}_{w}(\mu \frac{d{V}_{d}}{dy})$$

here, *A*_*w*_ is the wetted area of the droplet (*Aw* = *πr*^2^, where *r* corresponds to the wetted radius of the droplet), *μ* is the viscosity, *V*_*d*_ is the droplet fluid velocity, and *y* is the distance normal to the hydrophobic surface. The air-drag force is connected to the flow resistance when droplet is in motion in air ambient. It can be evaluated as ${D}_{a}\cong 1/2{C}_{d}{\rho }_{a}{A}_{c}{U}_{T}^{2}$, here, *C*_*d*_ corresponds to the drag coefficient^45^ and *U*_*T*_ represents the opposing air velocity, which is considered to be same of droplet translation velocity. Combining the forces in Eq. 1, the droplet rotational speed on the inclined surface yields:

4 $$\omega =\sqrt{(\frac{\frac{5}{2mR}(mg\,\sin \,\delta -\frac{24}{{\pi }^{3}}{\gamma }_{LV}f(\cos \,{\theta }_{R}-\,\cos \,{\theta }_{A})\,-{\mu }_{t}{A}_{w}\frac{\partial u}{\partial y})}{1+\frac{5}{4m}{C}_{d}{\rho }_{a}{A}_{c}R})}$$

When the particles are located on the surface, rolling droplet experiences the additional surface resistance due to the dust particles. Moreover, depending on the cloaking of the droplet fluid, the dust particles can be removed from the surface across the droplet wetted area. This increases the droplet mass and modifies the droplet dynamics on the surface. Hence, the droplet rolling is accompanied by the sliding on the surface. The droplet undergoes puddling due to gravity^13^. The puddling effect becomes minimum for small diameter droplets; hence, the droplet diameter smaller than the capillary length (${\kappa }^{-1}=\sqrt{\frac{{\gamma }_{LV}}{\rho g}}$, where *κ*^−1^ is the capillarity length*, γ*_*LV*_ is the surface tension*, ρ* is the density, and *g* is the gravitational acceleration) becomes like a spherical droplet during rolling on the surface. As the droplet diameter increases, puddling is resulted. The droplet pinning on the surface, due to contact angle hysteresis, alters the mass center of the fluid. This change in center of mass causes droplet wobbling on the surface, which affects the rotational dynamics of the droplet. Hence, the droplet with greater diameter than the capillarity length experiences bulging while causes a droplet puddling. However, the thickness (*h*) of the puddle (puddle height) is presented earlier^46^ and it is $\sqrt{2(1-cos\theta )\frac{{\gamma }_{LV}}{\rho g}}$, here *θ* corresponds to the contact angle. The translational velocity of the droplet can be assessed by the energy balance consideration. Since the formulism of the velocity for the droplet is given in the early work^11^, the brief description of the formulation is provided herein. Energy balance around the droplet yields^11^:

5 $${\rm{\Delta }}{E}_{Tot}-{\rm{\Delta }}{E}_{Diss}={\rm{\Delta }}{E}_{Kin}$$

here, *ΔE*_*tot*_ represents the droplet potential energy on the inclined surface. The total droplet potential energy becomes *ΔE*_*tot*_ = *mgΔh*, here *m* is the droplet mass, *g* represents the gravity, and *Δh* corresponds to the vertical distance. *E*_*Diss*_ corresponds to the droplet energy dissipation due to friction, adhesion, shear and air drag forces. The dissipated energy (*E*_*Diss*_) becomes *ΔE*_*friction*_ + *ΔE*_*deformation*_ + *ΔE*_*retention*_ + *ΔE*_*shear*_ + *ΔE*_*air-drag*_, here, *ΔE*_*friction*_ is the energy dissipated due to rate of fluid strain at the interface, *ΔE*_*deformation*_ corresponds to the energy dissipated due to the droplet puddling at which the center of droplet mass changes, *ΔE*_*retention*_ is related to the energy dissipated because of the droplet adhesion on the surface, and *ΔE*_*air-drag*_ is the energy dissipated due to the air-drag. The energy dissipated because of the volumetric deformation of the droplet during the puddling is ${\rm{\Delta }}{E}_{deformation} \sim {\forall }_{p}{\gamma }_{LV}(\frac{{D}_{{h}_{1}}-{D}_{{h}_{2}}}{{D}_{{h}_{1}}{D}_{{h}_{2}}})$, here ∀p represents the droplet volume, γ_LV_ is the surface tension and ${D}_{{h}_{1}}$ and ${D}_{{h}_{2}}$ are the droplet hydraulic diameter along the length scale *ΔL* on the surface due to puddling^11^. The energy dissipated due to the droplet adhesion is ${\rm{\Delta }}{E}_{adhesion} \sim \frac{24}{{\pi }^{3}}{\gamma }_{LV}Df\,{\rm{\Delta }}L(cos{\theta }_{R}-cos{\theta }_{A})$, here *θ*_*A*_ and *θ*_*R*_ are the dynamic advancing and receding angles of the droplet along the distance *ΔL*^11^. The shear loss at the interface between droplet-solid along the distance ${\rm{\Delta }}L$ is ${\rm{\Delta }}{E}_{shear} \sim {A}_{w}({\mu }_{t}\frac{d{V}_{d}}{dy}){\rm{\Delta }}L$, here *A*_*w*_ is the contact area (*A*_*w*_ = *πr*^2^, where *r* corresponds to the three-phase-contact radius), *μ*_*t*_ is the viscosity, *V*_*d*_ is the fluid velocity, and *y* is the vertical distance, which is normal to the surface^11^. The energy dissipated because of the air-drag is ${\rm{\Delta }}{E}_{air-drag}=\frac{1}{2}{K}_{L}m{U}_{T}^{2}$, here *K*_*L*_ represents the loss coefficient and the velocity in tangential direction (*U*_*T*_) can be calculated from the droplet angular speed (*ω*, Eq. 4) after incorporating the instantaneous hydraulic radius of the droplet on the surface, i.e. *D*_*H*_/2, is the instantaneous droplet hydraulic diameter. Hence, the translational velocity can be obtained incorporating the potential energy and energy dissipated due to droplet motion, i.e.^11^:

6 $$V=\sqrt{\begin{array}{l}2g[{\rm{\Delta }}Lsin\alpha -{\mu }_{f}{\rm{\Delta }}L-\frac{1}{mg}\frac{24}{{\pi }^{3}}{\gamma }_{L}Df{\rm{\Delta }}L(cos{\theta }_{R}-cos{\theta }_{A})\\ \,\,-\frac{4{\gamma }_{L}}{\rho g{\rm{\Delta }}L}(\frac{{D}_{{h}_{1}}-{D}_{{h}_{2}}}{{D}_{{h}_{1}}{D}_{{h}_{2}}})-\frac{1}{mg}{A}_{w}({\mu }_{t}\frac{d{V}_{f}}{dy}){\rm{\Delta }}L-\frac{1}{2g}{K}_{L}{U}_{T}^{2}]\end{array}}$$

Equation 6 can be incorporated predicting the droplet translational velocity on the surface.

**Figure 7:**
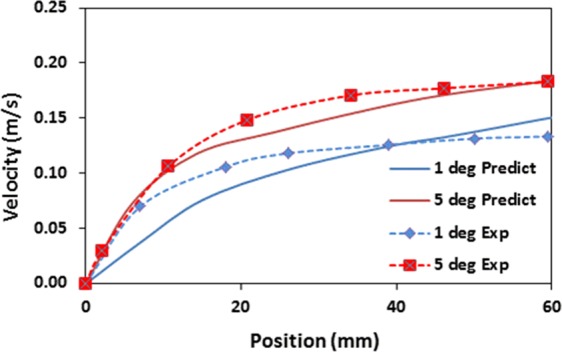
Droplet translational velocity predicted from analytical formulation and obtained from experiment with distance along inclined surface for two inclination angles. The droplet volume is 60 µL.

**Figure 8:**
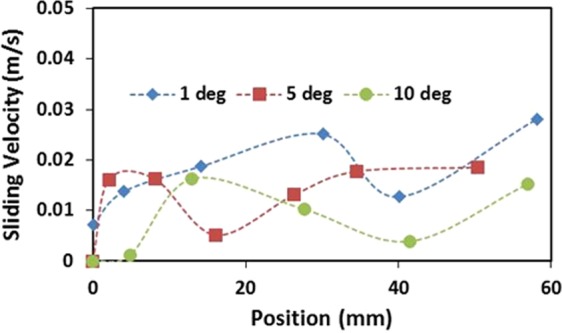
Sliding velocity of droplet along the inclined surface for various inclination angle of surfaces. The droplet volume is 60 µL.

Figure 7 depicts droplet translational velocity predicted from Eq. 6 and obtained from the experiments for 60 µL droplet and two inclination angles of the surface. The translational velocity is presented without the presence of the dust particles on the surface. Increasing inclination angle enhances the droplet translational velocity because of the gravitational acceleration. The droplet velocity increases along the short distance from the droplet motion is initiated. However, when the distance enlarges along the surface, the translational velocity rise becomes almost gradual. Hence, the initial acceleration of the droplet on the surface results in increased rise of the droplet velocity. When comparing the velocity predictions from Eq. 6 with those resulted from the high speed recording data, it is apparent that both results are in good agreement. Some small differences in both findings are related to the assumptions made in the translational velocity formulation and the experimental errors. Nevertheless, the difference is acceptably small. Since the droplet rolls and slides along the surface, the droplet sliding on the surface can be obtained through comparing the results of Eqs 1 and 6. In this case, the subtraction of the rotational tangential velocity (ω × R, ω is the rotational speed of droplet and *R* represents the instantaneous radius of the droplet. Here, ω is obtained from Eq. 1 and *R* is extracted from high speed recorded data along the surface) from the translational velocity (obtained from high speed records). Figure 8 shows the sliding velocity along the distance for various inclination angles of the surface and the droplet volume is 60 µL. The sliding velocity attains considerably smaller values than that of the translational velocity, i.e. the sliding velocity is almost 10% of the translational velocity. Consequently, the droplet mainly rolls on the inclined hydrophobic surface rather than sliding over the surface. Increasing inclination angle lowers the sliding velocity on the surface. This indicates that droplet acceleration due to increased inclination angle of the surface alters the droplet motion towards more rolling than rolling/sliding combination.

**Figure 9:**
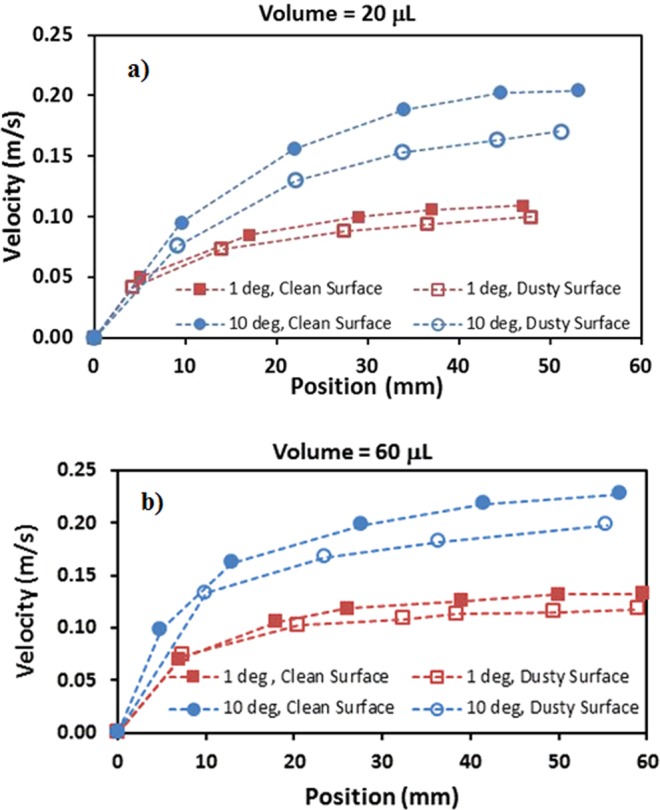
Droplet translational velocity with and without dust particles on the surface for two inclination angles: (**a**) droplet volume is 20 µL, and (**b**) droplet volume is 60 µL.

**Figure 10:**
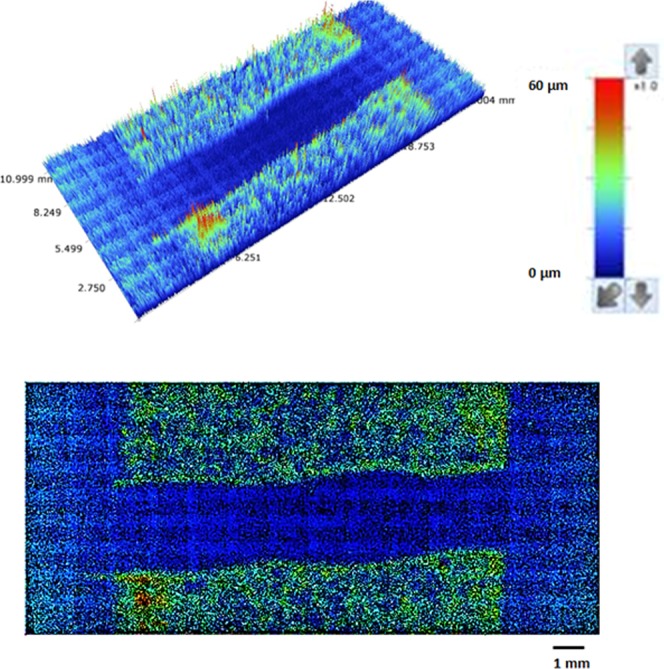
Droplet path on the dust and inclined hydrophobic surface. The droplet volume is 60 µL and inclination angle is 5°.

**Figure 11:**
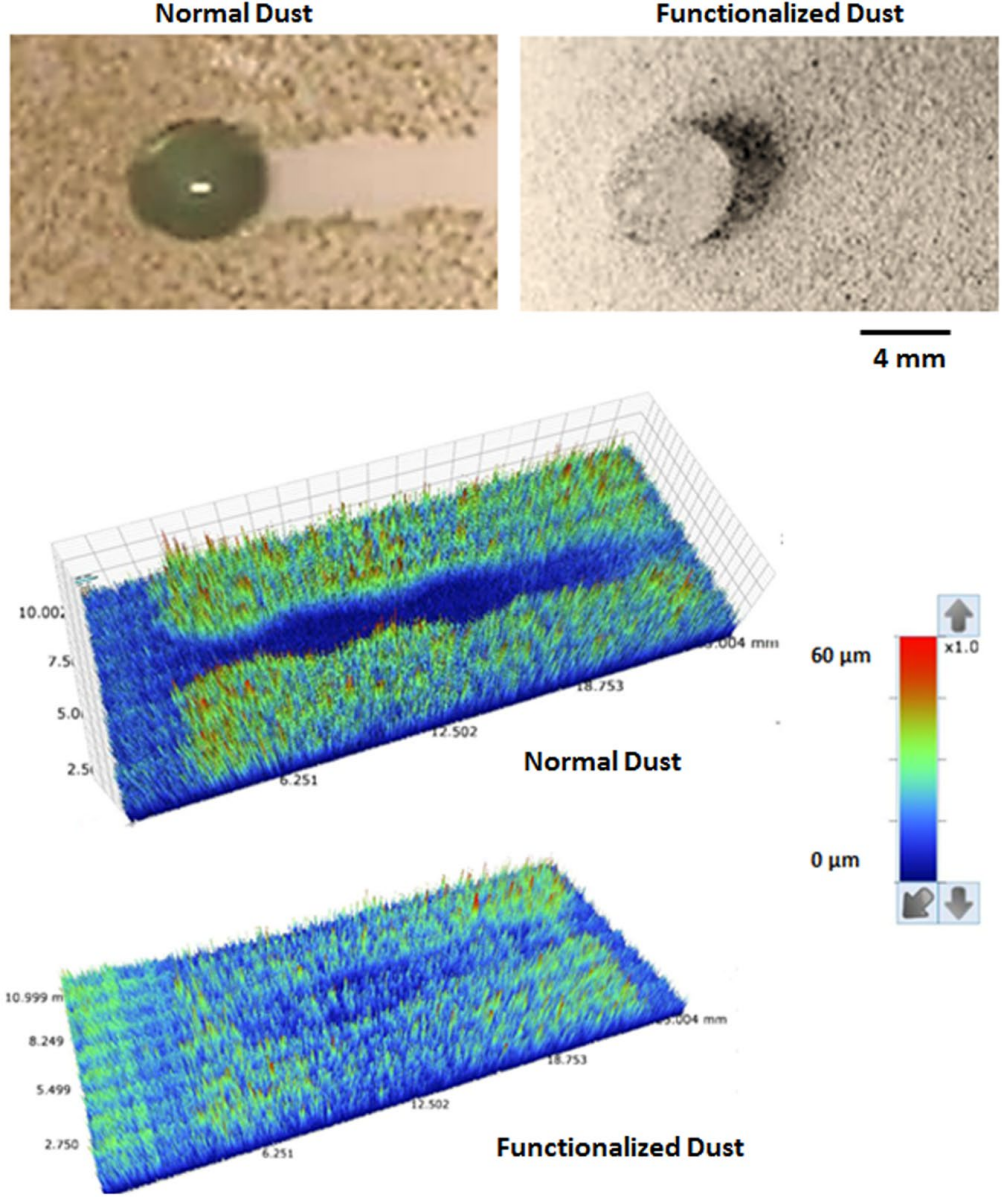
Rolling/sliding droplet on normal and functionalized dust deposited hydrophobic surface and 3D optical image of the surface. The droplet volume is 60 µL and inclination angle of hydrophobic surface is 1°.

**Figure 12:**
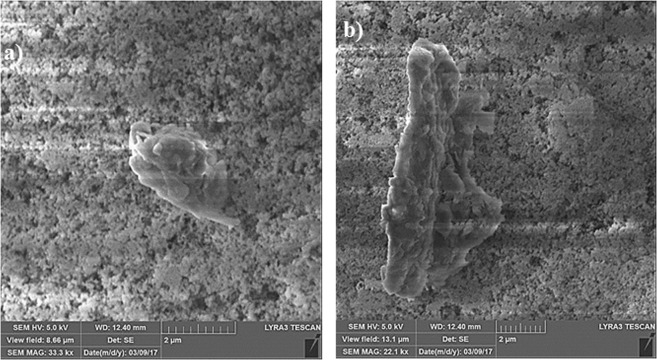
SEM micrographs of dust particle residues on the droplet path: (**a**) small and sharp edge dust particle, and (**b**) elongated shape dust particle.

**Figure 13:**
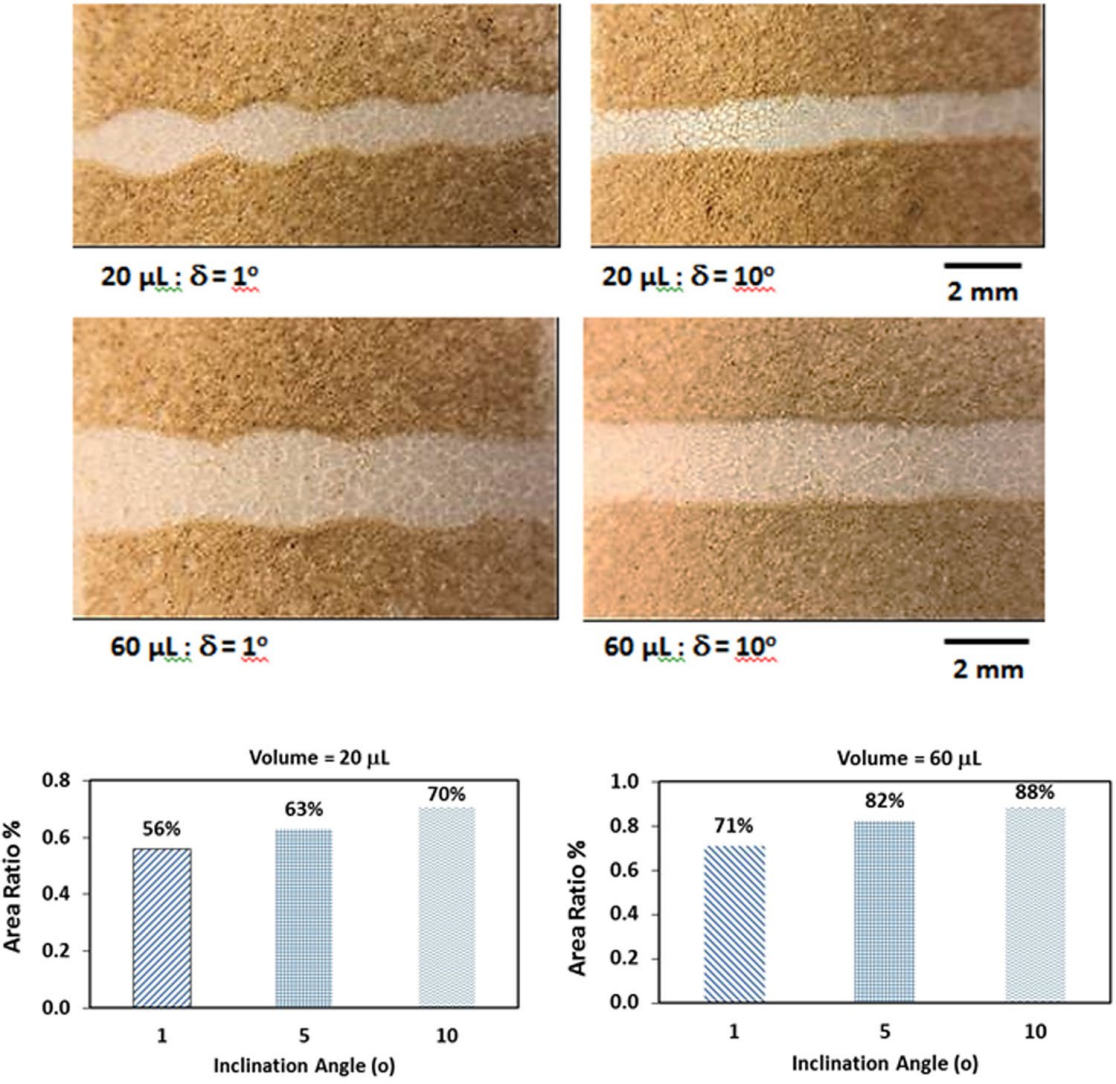
Optical image of droplet path on hydrophobic surface with presence of dust particles for two droplet size and two inclination angles. The ratio of area cleaned by droplet over the projected area (maximum wetting width × Sweeping length) is also shown for various inclination angles and two droplet volumes.

Figure 9a,b show translational velocity along the surface prior and after the dust particles deposition for two droplet volumes, respectively. The influence of the inclination angle is also included in Fig. 9a,b. Increasing droplet volume enhances droplet acceleration on the surface. This is because of the potential energy increase of the large size droplets; in this case, energy dissipated by the droplet during rolling/sliding becomes less than the potential energy increase due to the large droplet mass. However, enhancement of the translational velocity due to increase of droplet mass is not considerably high. Moreover, presence of the dust particles lowers the translational velocity, which is true for both droplet volumes. Although droplet picks up the dust particles during rolling/sliding and increases its mass, the trend of the translation velocity along the inclined surface remains almost similar to that corresponding to the undusted surface. However, for small volume droplet (20 µL, Fig. 9a) at high inclination angle (10°), the gap in between the droplet translational velocity due to dusty and undusted surfaces is slightly larger than that corresponding to the large size droplet (60 µL, Fig. 9b). Consequently, picking up the dust particles has greater influence on the droplet rolling/sliding motion for small volume droplet than that of the large volume droplet. On the other hand, during the droplet rolling/sliding, the particles are collected by the droplet. This situation can be observed from the path of the droplet on the dusty surface (Fig. 10). However, as the droplet rolls/slides along the surface, the dust particles located in the wetted region of the droplet are cloaked by the droplet fluid. Later, these particles mix with the fluid inside the droplet. Consequently, the dust particles are collected by the droplet during droplet transition over the surface. Further experiments are carried out to assess whether the only dust particles undergoing cloaking by the droplet liquid are picked up by the rolling droplet. Hence, some of the dust particles are functionalized to avoid droplet liquid spreading and cloaking. The dust particles are functionalized using trichloro(1 H,1 H,2 H,2H-perfluorooctyl) (PFOTS) by adopting the vapor deposition technique^47^. Figure 11 shows the residues of the functionalized and as received particles on the path of the droplet. It is evident that the residues of the functionalized dust particles is significantly more than that of the as received dust particles. Consequently, the main process involving for the particles removal from the surface by the rolling/sliding droplet is the cloaking by the liquid. The transition time corresponding to the droplet sweeping over the hydrophobic surface and wetted by the rolling/sliding droplet of 20 µL (it is the smallest volume considered with short wetted length) with inclination of surface at 10° (it is the largest inclination angle considered resulting in the highest transition velocity) is about 0.03107 s while the droplet fluid cloaking time for 20 µm dust particle (it is the largest size of the dust particle) is about 0.009175 s. The ratio of transition time over the cloaking time for the largest dust particle on the inclined hydrophobic surface is 3.386. Hence, the droplet fluid cloaks the dust particle during its sweeping on the inclined surface. This is true for the short wetting length of the droplet with the high translational velocity. In the case of the large volume droplet, the ratio of transition time over the cloaking time is about 6; hence, the droplet cloaks the dust particles during its transition on the surface and picks up the particles from the surface. Moreover, few of dust particles stay on the droplet path after the droplet rolls/slides over the dusty surface. The leftover of the dust particles (residues) on the droplet path are further investigated in terms of the shape and the elemental composition. Figure 12a,b show SEM micrograph of the dust particle residues on the droplet path while composition of the dust residues is provided in Table 1. In general, the dust particle residues are small in size and having sharp edges Fig. 12a,b. The elemental composition reveals that these particles compose of mainly silica (SiO_2_) and anhydrite (CaSO_4_). Hence, the sharp edges of the small size dust particles can partially penetrate into the functionalized silica particles coating and possibly anchoring to the coating. Hence, rolling/sliding droplet could not possibly pick up during its sweeping over the surface despite the fact that these particles are wetted by the droplet liquid. Nevertheless, the coverage area of dust residues is considerably small, which is almost 0.001% of the area covered by the droplet path. The mechanism of droplet wobbling is related to the droplet puddle height^46^ $(\sqrt{2(1-cos\theta )\frac{{\gamma }_{LV}}{\rho g}})$ and the contact angle hysteresis influence the droplet pinning on the surface (Eq. 2). Introducing the dust particles on the hydrophobic surface enhances the droplet pinning while lowering the droplet translational velocity (Fig. 9). Moreover, droplet wobbling and pinning enhancement on the dusty hydrophobic surface influences the droplet wetting diameter on the surface. Hence, the dust removal rate from the surface is influenced by the droplet wobbling and pinning on the surface. In this case, increasing pinning force and droplet puddle enhance the dust particles removal rate. Figure 13 shows the optical micrographs of droplet paths for 20 µL and 60 µL and two inclination angles of the hydrophobic surface. In addition, ratio of the area cleaned by the rolling/sliding droplet over the projected area of the dusty surface is provided with inclination angle for two droplet volumes. The projected area is considered to be equal to the maximum width of the droplet and total sweeping length. It should be noted that the maximum width of the droplet changes on the hydrophobic surface because of the droplet wobbling (Fig. 13). Increasing the angle of inclination of the hydrophobic surface causes droplet acceleration and the droplet wobbling on the surface during rolling/sliding motion reduces. This in turn lowers the droplet wetted width variation along the droplet sweeping length. Consequently, the ratio of the area cleaned by the droplet over the projected area (maximum wetting width × Sweeping length) remains high as the inclination angle increases. This behavior is true for all the droplet volumes considered in the present study. In the case of small inclination angle of the hydrophobic surface, droplet wobbling causes irregular pattern on the surface because of wetting width variation along the sweeping length, which is particularly true for large volume droplet (Fig. 13). The dust particles in the regions corresponding to the maximum and minimum wetting width of the droplet remain on the surface. Hence, small diameter droplet with large surface inclination angle remains favorable for the dust particles removal from surfaces.

## Conclusion

Water droplet behavior on the inclined hydrophobic surface is considered and dynamic characteristics of droplet rolling/sliding are examined. The dust particles removal from the inclined hydrophobic surface by rolling/sliding droplet is investigated and the dust particles removal mechanism is explored. We demonstrated that the predictions of the droplet translational velocity agree well with those resulted from the high speed recording data. The droplet rolling is the dominant mechanism on the hydrophobic surface and the ratio of the translational velocity over the sliding velocity is about 10. The dust particles have various sizes and shapes. The average dust particle size is about 1.2 µm and the dust particles compose of various elements. The particles are easily removed from the inclined surface by a water droplet. The main mechanism influencing the dust particle removal is the dust particles cloaking by water. The transition time of the droplet wetting on the hydrophobic surface is larger than the droplet fluid cloaking time of the dust particles. Hence, particles are cloaked by the water droplet along the wetted length of droplet and they are collected from the hydrophobic surface while facilitating the particles removal from the surface. Some residues of the dust particles are observed on the path of the droplet on the hydrophobic surface. In general, the dust residues are small in size with sharp edges and they probably anchor to the nano-size silica particles coating. The area covered cleaned by the rolling/sliding droplet on the surface highly depends on the droplet size and acceleration. Increasing droplet acceleration due to large inclination angle of the hydrophobic surface enhances the coverage area of the clean surface, which is more apparent for large volume droplets.
